# Assessment of Urban Ecosystem Health Based on Matter Element Analysis: A Case Study of 13 Cities in Jiangsu Province, China

**DOI:** 10.3390/ijerph14080940

**Published:** 2017-08-21

**Authors:** Xuefeng Xie, Lijie Pu

**Affiliations:** 1 School of Geographic and Oceanographic Sciences, Nanjing University, Nanjing 210023, China; xiexuefeng2008@126.com; 2 The Key Laboratory of the Coastal Zone Exploitation and Protection, Ministry of Land and Resources, Nanjing University, Nanjing 210023, China

**Keywords:** urban ecosystem health assessment, analytic hierarchy process, limiting factors, obstacle degree

## Abstract

Urban public health is an important global issue and receives public concern. The urban ecosystem health (UEH) indicator system was constructed with 27 assessment indicators selected from vigor, organization, resilience, service function, and population health, then the matter element analysis (MEA) and analytic hierarchy process (AHP) weighting method were used to assess the UEH of each city in Jiangsu Province during the period of 2000–2014. The results show that the overall ecosystem health status of each city shows continuous improvement. The UEH status of each city gradually transferred from poor, general, and medium condition to good and excellent condition. From the perspective of spatial distribution, the city’s UEH showing a steady status after increasing for 10 years, and their spatial variations have gradually reduced. The UEH status in Southern Jiangsu and Central Jiangsu was better than that of Northern Jiangsu Province. From each component point of view, the vigor, resilience, and population health of each city in Jiangsu Province showed a trend of continuous improvement, while the organization and service function first increased and then decreased. The common limiting factors of UEH in Jiangsu Province were Engel’s coefficient of urban households, number of beds of hospitals, health centers per 10,000 people, and total investment in the treatment of environmental pollution as percent GDP. These results help decision makers to make suitable decisions to maintain the UEH of each city in Jiangsu Province.

## 1. Introduction

Urban ecosystems are most strongly affected by human activities, especially in some rapid development regions [1,2]. In recent years, ecological environmental capacity that maintains the economic development, human health, and even social sustainable development have sharply reduced due to the acceleration of the urbanization process and the deterioration of the organization and service functions of urban ecosystems [3,4]. Thus, the assessment of UEH has gradually became a scientific topic and received public concern in the past years. Urban ecosystem health (UEH) is derive from the concept of ecosystem health and describes a state in which an urban ecosystem maintains its integrity and health to continue supplying eco-services to humans maintaining a healthy state [5,6,7]. Recently, the research on ecosystem health was mainly relate to the exploration of concept and connotation [8,9,10], the design of an indicator system [11,12], and the establishment and application of mathematical models [13,14,15].

Due to complexity of urban ecosystem, it is a tough task to construct an indicator system that includes both natural and social factors in the assessment of UEH [15]. A number of indicator systems have been proposed for the assessment of UEH conditions according to different understandings of the concepts of UEH, such as the Vigor-Organization-Resilience framework [16], the Natural-Economic-Social framework [17], the Pressure-State-Response framework [18], etc. As a highly complex social-economic-natural integrated system, the assessment of UEH should also take service functions and population health into consideration [19,20]. The Vigor-Organization-Resilience-Service-Function-Population health framework was based on multiple indicators selected from natural, economic, and social subsystems that comprehensively reflected the different dimensions of the urban ecosystem and can better assess the status of UEH.

Currently, many mathematical models (such as emergy analysis [13,21], set pair analysis [21], the fuzzy synthetic assessment model [22], the maximum information entropy method [23], material flow analysis [24], matter-element extension model [14], and the catastrophe progression model [25]) have applied to assess the ecosystem health. Although these methods focused on the characteristics of UEH and have played an important role in promoting the research of UEH, the complicated computation processes, and the information omissions in the process of calculation limited the application of these methods [26,27]. What is more, some assessing methods (such as fuzzy synthetic assessment, catastrophe progression model, etc.) often divided the ecosystem health artificially into several grades according to the comprehensive value, which could not identify the membership degree between the single index, general index, and evaluation ranks [28]. Matter element analysis (MEA) can greatly expand the range of research to reveal more differentiation information and get the state of individual indicators through the calculation of indicator correlation function, then get the comprehensive level of multiple targets [29]. Additionally, MEA can show the intermediate transformation status of comprehensive evaluation results and improve the objectivity and accuracy of grade determination [30]. Simultaneously, the diagnosis of obstacle factors can help the sustainable ecological management at the regional scale [31].

In this paper, considering the special characteristics of UEH, a framework indicator system and related indicators were selected from vigor, organization, resilience, services function, and population health. Meanwhile, the MEA and analytic hierarchy process (AHP) methods were applied to assess the UEH of Jiangsu Province. Then, the obstacle degree was used to analysis the limiting factors of UEH in Jiangsu Province. The last section presents some discussion and conclusions.

## 2. Materials and Methods

### 2.1. Study Site

**Figure 1:**
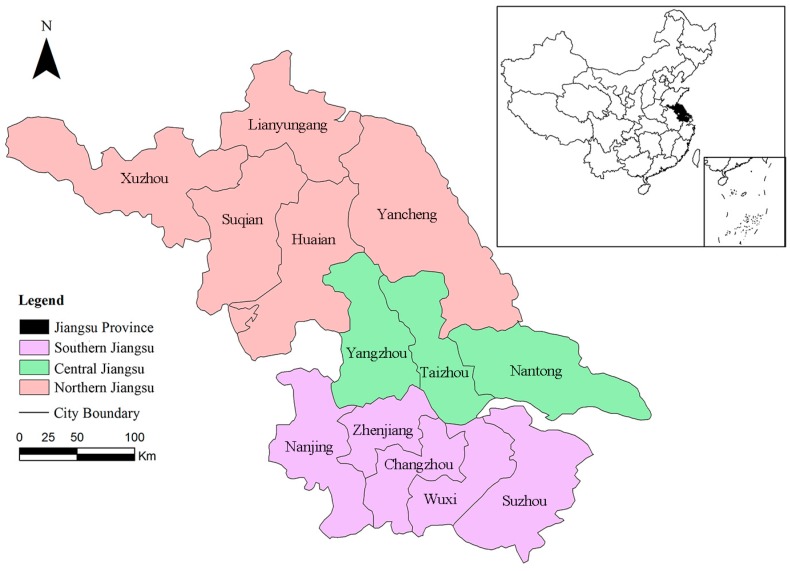
Location of the study area.

Jiangsu Province is an important economic zone in the Yangtze River delta located in East China and extending from 116°18′ E to121°57′ E and from 30°45′ N to 35°20′ N with 13 prefectural cities. Its capital is Nanjing (Figure 1). Jiangsu Province covers a total area of 10.26 × 10^4^ km^2^ with a population of 79.2 million. By 2014, Jiangsu Province had the highest Gross Domestic Product (GDP) per capita ($13,328) in China. However, with rapid urbanization in last three decades, the environmental pressures of this region have significantly increased, such as the deterioration of water quality, air pollution, energy shortages, and traffic congestion. According to differences in economic development, Jiangsu Province has usually been divided into Southern Jiangsu (SJ, including Suzhou, Wuxi, Changzhou, Zhenjiang, and Nanjing, which is the most developed region in Jiangsu Province), Central Jiangsu (CJ, including Nantong, Taizhou and Yangzhou, which the economy is relatively Good in Jiangsu Province), and Northern Jiangsu (NJ, including Yancheng, Lianyungang, Huai’an, Suqian, and Xuzhou, which the economy is relatively backward in Jiangsu Province). The characteristics of surveyed cities in Jiangsu Province in 2014 are shown in Table 1.

### 2.2. Design of Indicator System

Establishing a holistic indicator system is the key point of UEH assessment. In general, indicators are selected based on the principle of data acquisition, regionality, scientific, representative, objectivity, and early warning [12,15]. The Vigor-Organization-Resilience-Service-Function-Population health frameworks is an extension of Vigor-Organization-Resilience framework, and taken ecosystem service and public health into consideration [14,23]. The UEH assessment indicator system of this paper is constructed based on the Vigor-Organization-Resilience-Service-Function-Population health framework. The indicators were selected from natural, economic, and social dimensions of the urban ecosystem according to the current situation of each city in Jiangsu Province and refers to an extensive literature review [3,12,14,15,23,25,32]. Table 2 shows that the urban ecosystem is divided into five components: (a) Vigor, which reveals a city’s vitality and metabolic. We selected seven indicators X1 (Per capita GDP), X2 (GDP growth), X3 (Energy consumption per 10,000 CNY of GDP), X4 (Per capita annual disposable income of urban households), X5 (Actual use of foreign capital), and X6 (Green covered area as of completed area) to represent the productivity and resource consumption of the region. (b) Organization, which reveals the diversity of configuration of the natural, economic and social structure in urban areas. X7 (Population density of urban area), X8 (Natural growth rate of population), X9 (Tertiary industry accounted for the proportion of GDP), X10 (Proportion of tertiary industry employment), X11 (Fiscal revenue accounted for the proportion of GDP), and X12 (Growth rate of total investment in fixed assets) were selected to reveal the ecosystem social and economic organization. (c) Resilience, which ensuring the sustainable development of urban ecosystem, reflecting a kind of systematic self-regulation. However, the resilience of urban ecosystem mainly depends on the humanity management activity. Thus, X13 (Attainment rate of the industrial wastewater discharged), X14 (Urban wastewater treatment rate), X15 (Common industrial solid wastes comprehensively utilized), and X16 (Total investment in the treatment of environmental pollution as percent GDP) were adopted to indicate the self-regulation of urban ecosystem. (d) Service function, which reveals the function of urban ecosystem that provide the carrier of human production and life. X17 (Proportion of days of air quality equal to or above grade II in the whole year), X18 (Urban environmental noise), X19 Per capita area of parks and green land), X20 (Per capita urban residential area), X21 (Per capita area of paved roads in city), X22 (Number of public transportation vehicles per 10,000 population in city), and X23 (Registered urban unemployment rate) were selected to reveal the capacity of unban ecosystem for mankind to exist and live in. (e) Population health, which is definitely the core issue of urban ecosystem health, as mankind is the subject in an urban ecosystem. We selected four indicators: X24 (Engel’s coefficient of urban households), X25 (Under 5 mortality rate), X26 (Number of beds of hospitals and health centers per 10,000 population), and X27 (Number of students’ enrollment of regular institutions of higher education per 10,000 population) to represent people’s physical and mental health or the important factors affecting the well-being and health of humankind.

### 2.3. Matter Element Analysis

The matter element analysis can reveal more extensively objective information and solve complex problems in multi-factor assessment, which is incompatible [30]. The matter element analysis results are obtained by the correlation coefficient, which were calculated by the single indicator and standard grade.

The basic procedure of matter element analysis for UEH can be summarize as the following steps [33]:

● Step 1: Construct the fuzzy matter-element.

During matter element analysis, the UEH *N*, its character *C* and quantity value x are expressed as *R* = (*N*, *C*, *x*). This combination is known as the matter element. If UEH *N* needs to be described by *n* characters *C*_1_, *C*_2_, …, *C_n_* and corresponding quantity values *x*_1_, *x*_2_, …, *x_n_*, then it can be called an *n*-dimension matter element, which is expressed by the following matrix:

(1) $$R=\left|\begin{array}{ccc} N & C_{1} & x_{1}\\ & C_{2} & x_{2}\\ & \vdots & \vdots \\ & C_{n} & x_{n} \end{array}\right|=\left|\begin{array}{c} R_{1}\\ R_{2}\\ \vdots \\ R_{n} \end{array}\right|$$

● Step 2: Determine classical domain and joint domain.

The classical domain matter element matrix of UEH can be expressed as,

(2) $$R_{oj}=\left(N_{oj},C_{i},x_{oij}\right)=\left(\begin{array}{ccc} N_{oj} & C_{1} & x_{o1j}\\ & C_{2} & x_{o2j}\\ & \vdots & \vdots \\ & C_{n} & x_{oij} \end{array}\right)=\left(\begin{array}{ccc} N_{oj} & C_{1} & \left(a_{o1j},b_{o1j}\right)\\ & C_{2} & \left(a_{o2j},b_{o2j}\right)\\ & \vdots & \vdots \\ & C_{n} & \left(a_{oij},b_{oij}\right) \end{array}\right)$$

In this matrix, *R_oj_* is classical domain matter element, *N_oj_* is the jth grade of UEH (*j* = 1, 2, …, m), *C_i_* is the *ith* character of the *jth* grade, and *x_oij_* is the quantity value of *N_oj_* with respect to *C_i_*: i.e., the classical domain describing the corresponding characteristics of each grade (*a_oij_*, *b_oij_*).

The joint domain matter element matrix of UEH can be expressed as,

(3) $$R_{p}=\left(N_{p},C_{i},x_{pi}\right)=\left(\begin{array}{ccc} N_{p} & C_{1} & x_{p1}\\ & C_{2} & x_{p2}\\ & \vdots & \vdots \\ & C_{n} & x_{pn} \end{array}\right)=\left(\begin{array}{ccc} P & C_{1} & \left(a_{p1},b_{p1}\right)\\ & C_{2} & \left(a_{p2},b_{p2}\right)\\ & \vdots & \vdots \\ & C_{n} & \left(a_{pn},b_{pn}\right) \end{array}\right)$$

In this matrix, *R_p_* is joint domain matter element; *N_p_* is all grades of UEH; *x_pi_* is the quantity value of *R_p_* with respect to *C_i_*—joint domain (*a_pi_*, *b_pi_*), here requires *x_oij_* ∈ *x_pi_*.

According to the extension of UEH, UEH status can be classified into Excellent, Good, Medium, General, and Poor. The classical domain and joint domain of UEH was determined mainly based on the “Planning of ecological civilization construction in Jiangsu Province” [34], the average value of thirteen prefectural cities, and previous academic research [12,14,25,32]. Meanwhile, this paper adopted the expert evaluation method to make sure that the standards on the value range of the UEH indicators are scientific and reasonable. We selected five experts from the Statistics Bureau of Jiangsu Province (for evaluate X11 and X12) and the Civil Affairs Bureau of Jiangsu Province (for evaluate X8 and X25) to determine the standard of X8, X11, X12, and X25 through the questionnaire survey. The grades of UEH indicators are shown in Table 3, which is the basis for calculating correlation function value and comprehensive correlation degrees.

● Step 3: Determine matter elements to be rated.

The matter elements to be rated can be expressed as,

(4) $$R_{k}=\left(N_{k},C_{i},x_{pi}\right)=\left|\begin{array}{ccc} N_{k} & C_{1} & x_{p1}\\ & C_{2} & x_{p2}\\ & \vdots & \vdots \\ & C_{n} & x_{pn} \end{array}\right|$$

In this matrix, *N_k_* is the matter to be rated (*k* = 1, 2, …, *n*) and *x_i_* is the quantity value of *N_k_* with respect to *C_i_*, i.e., the actual data of each character.

● Step 4: The UEH evaluation indicator correlation function *K*_(*Ci*)*j*_ can be expressed as,

(5) $${K_{\left(C_{i}\right)}}_{j}=\left\{ \begin{array}{l} \frac{-\rho_{ij}(v_{i},x_{oij})}{\left|x_{oij}\right|},v_{i}\in v_{o}\\ \frac{\rho (v_{i},x_{oij})}{\rho_{pi}(v_{i},x_{pi})-\rho (v_{i},x_{oij})},v_{i}\notin v_{o} \end{array}\right.$$

In this equation:

(6) $$\left\{ \begin{array}{l} \rho_{ij}(v_{i},x_{oij})=\left|v_{i}-\frac{1}{2}(a_{oij}+b_{oij})\right|-\frac{1}{2}(b_{oij}-a_{oij})\\ \rho_{pi}(v_{i},x_{pi})=\left|v_{i}-\frac{1}{2}(a_{pi}+b_{pi})\right|-\frac{1}{2}(b_{pi}-a_{pi}) \end{array}\right.$$

● Step 5: Calculate weight co-efficient.

In order to define the priority of various indicators, a certain weight is assigned to each of them. The weight co-efficient was calculate by the analytic hierarchy process (AHP) method, which involves four steps [35,36]: (1) structuring the decision problem into a hierarchical model, (2) making pair-wise comparisons and obtaining the judgmental matrix, (3) individual priorities and consistency of comparisons, and (4) aggregation of individual priorities. The weight of all twenty-seven indicators were calculated by AHP method and was shown in Table 2.

● Step 6: Calculate the synthetically correlation degree and determine the matter level.

The synthetically correlation degree *K_j_*(*N_k_*) can be expressed as,

(7) $$K_{j}(N_{k})=\sum_{i=1}^{n}\omega_{i}k_{j}(x_{i})$$

In this formula, *K_j_*(*N_k_*) is the synthetically relational degree, *k_j_*(*x_i_*) is the single correlation degree, and *ω_i_* is the weight of each indicators. According to the maximum subordination principle in fuzzy mathematics, if *K_jk_*= max (*K_j_*(*N_k_*)), (*j* = 1, 2, …, *n*), then the *N_k_* belongs to the *j*th grade of UEH [31].

### 2.4. Diagnosis of UEH Limiting Factors

Diagnosis of the main limiting factors that affect the health of urban ecosystem can provide theoretical reference and support for the government to develop differentiated and targeted policies and measures [37]. Limiting factors were calculated by factor contribution degree, indicator deviation degree, and obstacle degree. The factor contribution degree (*V_j_*) represents the degree of influence of the single factor on the overall objective: the weight of the single factor to the total objective (*w_i_*·*w_ij_*). Deviation of the index (*x_ij_*) indicates the gap between the individual index and the UEH target, namely the difference between the standardized value of the individual index and 1. Obstacle degree (*Yi*, *yi*) indicates the influence of the *i* year classification index and single index on the urban ecosystem health and is the objective and result of the diagnosis of urban ecosystem health disorder. The formula is as follows [31]:

(8) $$x_{ij}=1-X\prime_{ij}$$

(9) $$y_{i}=x_{ij}\times V_{j}/\sum_{j=1}^{n}\left(x_{ij}\times V_{j}\right)\times 100\%$$

(10) $$Y_{i}=\sum y_{i}$$

### 2.5. Data Sources and Processing

Here, the research data were selected from the statistical yearbooks of Jiangsu Province (2001, 2006, 2011, and 2015) [38,39,40,41] and the statistical yearbook and environmental quality bulletin of Jiangsu Province (2001, 2006, 2011, and 2015) [42,43,44,45]. The data were processed using Microsoft Excel 2007 (Microsoft, Redmond, WA, USA) and SPSS Statistics 22.0 (IBM, Armonk, NY, USA), and the maps were drawing by the Origin 8.5 (OriginLab, 2009, Northampton, MA, USA) and ArcGIS 10.3 (ESRI, 2013, Redlands, CA, USA).

## 3. Results and Analysis

### 3.1. Results and Analysis of Urban Ecosystem Health

The correlation degree of the indicators of the UEH were calculated based on Equations (5)–(7). This paper uses the correlation degree of the X1 (Per capita GDP) of Nanjing as an example and analyzes the parameters of the computation process. The actural value of X1 in Nanjing was 10.773. Using Equations (5)–(7), the single index’s correlation degree was calculated as follows: *k*_1_(X1) = −0.487, *k*_2_(X1) = −0.423, *k*_3_(X1) = −0.231, *k*_4_(X1) = 0.061, and *k*_5_(X1) = −0.117. According to maximum membership principle, the X1 in Nanjing was classified as grade IV (Good). The UEH grades of other indicators of thirteen cities in 2014 were similarly diagnosed and are shown in Table 4.

By using Equations (2)–(7), the comprehensive correlation degree of UEH in Nanjing in 2014 was obtained as follows: *k*_1_(*N*) = −0.4549, *k*_2_(*N*) = −0.3786, *k*_3_(*N*) = −0.2670, *k*_4_(*N*) = −0.1723, and *k*_5_(*N*) = −0.1439. Thus, the UEH status of Nanjing in 2014 was classified as excellent grade. The UEH grades of other cities in 2000, 2005, 2010, and 2014 were similarly diagnosed, and are presented in Table 4.

As shown in Table 5, the overall ecosystem health status of each city in Jiangsu Province shows continuous improvement. In 2000, the UEHs were mainly of general status and then gradually rose to excellent status in 2014. During 2000–2005, the UEH status of each city in Jiangsu Province has improved significantly. Generally speaking, the assessment rating of excellent, good, and medium urban areas increased by 2.54 × 10^4^ km^2^, 0.66 × 10^4^ km^2^, and 5.5 × 10^4^ km^2^, accounting for 24.8%, 6.5%, and 54% of the total areas of Jiangsu Province, respectively (Table 6). During 2005–2010, the UEH status of each city continued to improve (except Yancheng), areas below the medium level gradually disappeared, and the excellent and good urban areas reached 8.6 × 10^4^ km^2^ and 1.7 × 10^4^ km^2^, accounting for 83.5% and 16.5% of the total areas (Table 6). The UEH status gradually stabilized during 2010–2014, and most of the UEH level of each city in 2014 were consistent with 2010: only Lianyungang reduced from excellent to good status.

### 3.2. Temporal and Spatial Distribution of Urban Ecosystem Health

**Figure 2:**
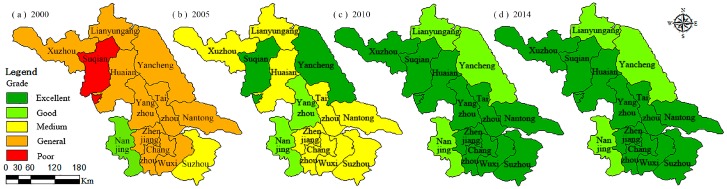
UEH status of Jiangsu province within different years: (**a**) 2000, (**b**) 2005, (**c**) 2010, (**d**) 2014.

As shown in Figure 2, in 2000, the UEH of Nanjing and Suzhou, which belongs to SJ, had a good and medium status, respectively, while Suqian in NJ had a poor status, and the rest of the cities of Jiangsu received general status. The overall UEH status of SJ is better than CJ, and NJ is the worst.

In 2005, the UEH status of NJ improved significantly. Suqian and Yancheng received excellent status, which was obviously superior to that in SJ and CJ. The UEH status gradually stabilized during 2010–2014. SJ, CJ and NJ (except Yancheng) all had excellent status, while the UEH status of Lianyungang reduced from excellent to good. This is mainly due to two elements: the rapid development of economic in Jiangsu Province, which gradually improved people’s living standards, environmental quality, and social security, and the economy of SJ developed rapidly due to its proximity to Shanghai, and the infrastructure facilities comparatively matured, while the economic development of NJ is relatively backward, resulting in lower UEH level than SJ. After more than 10 years of rapid development, the differences in infrastructure, social security and people’s living standard of Jiangsu Province has gradually reduced, which leads to the gradual reduction of the UEH level of the ecosystem.

### 3.3. The Contribution Value to Overall Health Index from Each Component

**Figure 3:**
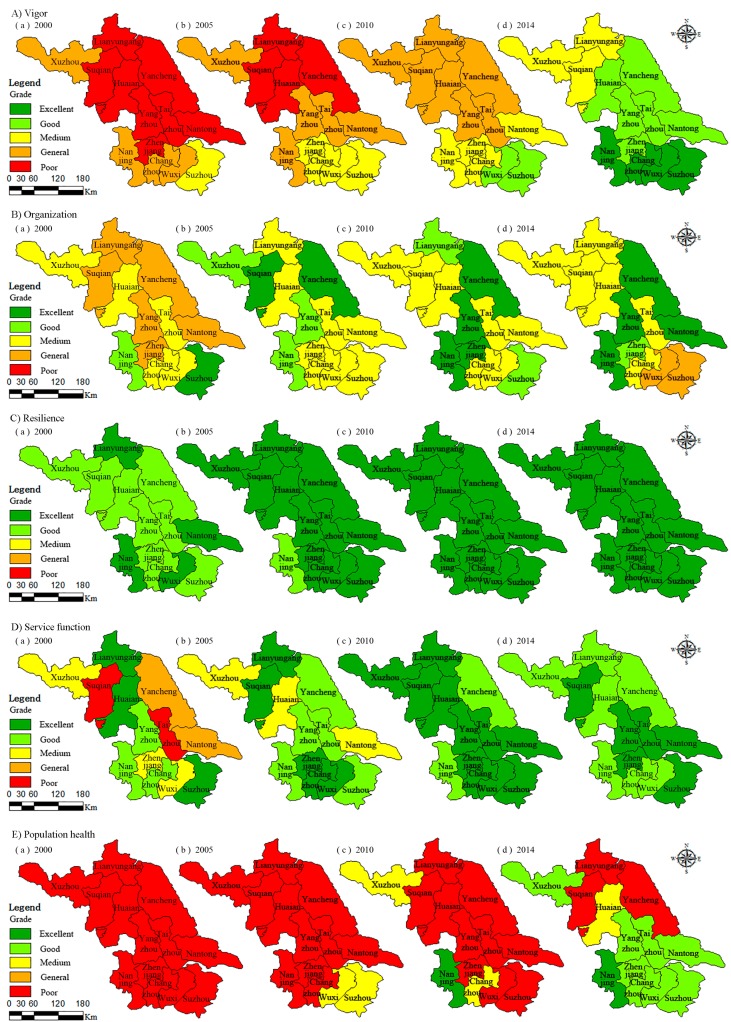
Each component ((**A**) Vigor, (**B**) Organization, (**C**) Resilience, (**D**) Service function, and (**E**) Population health) health of UEH in Jiangsu province within 2000, 2005, 2010, and 2014.

As shown in Figure 3A, the vigor of ecosystem of each city in Jiangsu Province is improving during 2000–2014, and the UEH level is roughly in SJ > MJ > NJ. The vigor of ecosystem in 2000 is poor, in which Suzhou is at the medium level, Nanjing, Changzhou, Wuxi and Xuzhou in the general level and the rest of the region are in poor level. The vigor of ecosystem in 2014 gained greatly improved compared with 2000. This is mainly owing to the improvement of Per capita GDP, Per capita annual disposable income of urban households, and the decrease of energy consumption, especially in SJ. Take Nanjing for example, the Per capita GDP, Per capita annual disposable income of urban households, and the energy consumption per 10,000 CNY of GDP in 2000 is 1.89 × 10^4^ CNY, 0.82 × 10^4^ CNY and 1.01, respectively. While in 2014, the Per capita GDP, Per capita annual disposable income of Nanjing has increased to 10.77 × 10^4^ CNY and 4.26 × 10^4^ CNY, whereas energy consumption reduced to 0.55.

The ecosystem organization were significantly different among different cities in Jiangsu Province during 2000–2014 (Figure 3B). Suzhou and Nanjing were excellent and good, while Changzhou, Wuxi, Taizhou, Huai’an, and Xuzhou were medium, and the rest of the cities were general in 2000. By 2014, the UEH level of Nanjing, Yangzhou, Yancheng, Nantong, Zhenjiang, Lianyungang, and Suqian improved, while Suzhou and Wuxi were reduced to the general level. This phenomenon was mainly caused by two reasons. First, the increase of tertiary industry accounted for the proportion of GDP, proportion of tertiary industry employment, and total investment in fixed assets, which resulted in improvement of the urban ecosystem organization in CJ and NJ. Second, the increase in population density in some developed regions such as Suzhou and Wuxi caused increased social and environmental pressure, and industrial transfer led to the decrease of the growth rate of fixed assets investment, which degraded the ecosystem organization in SJ.

As presented in Figure 3C, the resilience of UEH of each city in Jiangsu province were in good condition throughout the past 15 years because of the high rate of waste disposal and environmental protection investment. The attainment rate of the industrial wastewater discharged, urban wastewater treatment rate, common industrial solid wastes comprehensively utilized were both above 90%, and the total investment in the treatment of environmental pollution as percent GDP were basically maintained at 3% during 2000–2014.

The level of ecosystem services in the cities in Jiangsu Province fluctuated (Figure 3D), of which the ecosystem service function in 2000 was the worst. By 2010, the ecosystem service function of each city greatly improved compared with 2000. Nanjing and Yancheng were good, while the rest were all excellent. After that, the ecosystem service function of Changzhou, Wuxi, Xuzhou, Huai’an, and Lian Yungang reduced to good. This was probably due to the emission of waste gas from factories and automobiles, gradually making the air pollution serious. What is more, the increase of vehicles and the construction of urban areas led to the gradual increase of environmental noise. This led to the decrease of urban ecosystem function maintenance.

As depicted in Figure 3E, the level of population health significantly increased in some cities during 2000–2014. As urban public infrastructure was still not perfect, the population health status of Jiangsu Province was poor in 2000. By 2014, with the development of economy, the public infrastructure in the developed areas has gradually improved, which resulted in good and excellent levels of population health in SJ and MJ, whereas the NJ was still poor.

### 3.4. Limiting Factors Analysis of Urban Ecosystem Health

**Figure 4:**
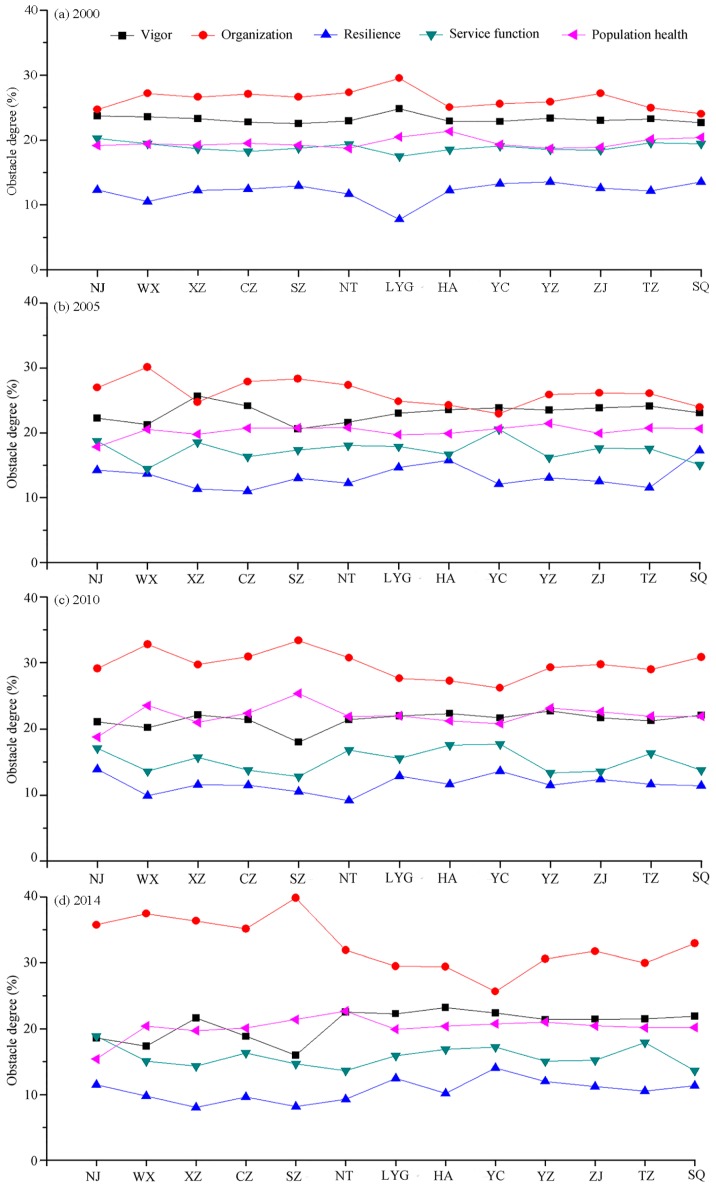
Obstacle degree of each component in different city from 2000 to 2014. (Line with different colors represents the obstacle degree values of different components of urban ecosystem health; NJ: Nanjing; WX: Wuxi; XZ: Xuzhou; CZ: Changzhou; SZ: Suzhou; NT: Nantong; LYG: Lianyungang; HA: Huai’an; YC: Yancheng; YZ: Yangzhou; ZJ: Zhenjiang; TZ: Taizhou; SQ: Suqian).

Obstacle degree of each component in different cities during 2000 to 2014 are shown in Figure 4. In general, the obstacle degree to urban ecosystem health varies with different components and was successively organization > vigor > population health > service function > resilience during 2000–2014. In view of the dynamic change of the obstacle degree from 2000 to 2014 (Figure 4), the obstacle degree of urban ecosystem organization gradually increased, and urban ecosystem vigor, resilience, and service function fluctuating declined. However, the obstacle degree of population health remained stable. This is attributed to the establishment of a resource-saving and environmentally friendly society in Jiangsu Province during the last 15 years and as a result in decrease of energy consumption and improved urban environment (air quality, parks, green lands, etc.).

**Figure 5:**
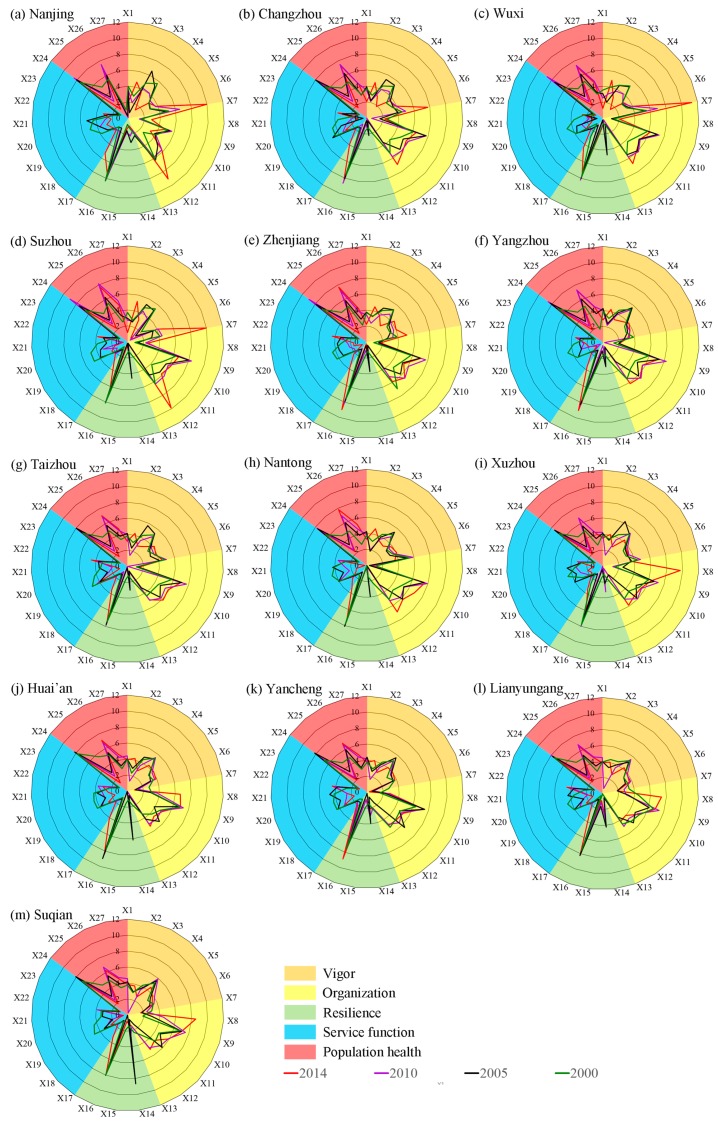
Obstacle degree of each indicator in different city from 2000 to 2014 (Different background colors represents different components of urban ecosystem health, lines with different colors represents obstacle degree values of each indicators with different years).

As depicted in Figure 5, obstacle degree of each indicator in different city showed a certain similarity and divided into two categories. The first is the rapid urbanized areas, such as SJ. These areas were characterized by high population density, lower growth rate of total investment in fixed assets, and the degree of limiting factors of these indicators increased during 2000–2014 (Figure 5). The other is the relatively slow urbanization areas, such as MJ and NJ. These cities usually had a high obstacle degree of natural growth rate of population and tertiary industry accounted for the proportion of GDP. Simultaneously, all cities had some common limiting factors, such as high Engel’s coefficient of urban households and a lack of hospitals and health centers (Figure 5).

## 4. Discussion

A series of indicator systems were proposed to assess the UEH conditions, but each framework and model is a major organizing paradigm and different understanding of the concept of UEH when assess the UEH conditions. Liu et al. (2009) proposed emergy theory and vigor-structure-resilience-function maintenance framework to assess the UEH of Baotou, China [13]. Liu et al. (2016) developed the pressure-state-response framework and comprehensive index methods to evaluate the ecosystem health of Loess Plateau [18]. To our knowledge, urban ecosystem health was affect by natural, economic and social factors [23,46]. The Vigor-Organization-Resilience-Service-Function-Population health framework used in this research was based on multiple indicators selected from natural, economic, and social subsystems, which comprehensively reflected the different dimensions of urban ecosystem and also took public health into consideration [47]. It can better assess the status of UEH compared with other similar studies [12,16,18]. Similarly, the selection of indicators also had a great effect on assessed the status of UEH. For example, our results revealed that the UEH conditions of Nanjing, Wuxi, and Suzhou were excellent in 2014, while the result of Zhao and Chai (2015) showed that both were sub-healthy in status in 2013 [23]. This was primarily attributed to the differences in the selection of indicators between them. Zhao and Chai (2015) selected 12 indicators to characterize the UEH that mainly reflects the vigor, organization, and resilience of the urban ecosystem, while our research proposed a relative holistic indicator system contains 27 indicators that also reflect urban ecosystem services and population health [23]. However, the indicators we selected still have some limitations due to the availability and consistency of data. The indicators we used here could not fully reflect the natural health of the urban ecosystem, making the result seems as if the evaluated factors are related with the economical progression more than ecosystem natural health because we have not considered the contamination conditions of water and soil or the richness and diversity of plants and animals in the study area. The ecosystem health status of each city revealed that economic status is not the only factors that affects UEH: the natural and social indicators we selected also play an important role in UEH. For instance, the UEH status is successively Nanjing > Nantong > Suzhou > Yangzhou > Wuxi in 2014 (Table 5), while the economic level of Suzhou and Wuxi are significantly superior to Nantong and Yangzhou. Although not all indicators involved can help to realize a better urban ecosystem, they can still provide a reference for decision makers to make specific regulations to maintain the UEH of each city in Jiangsu Province.

The application of different methods can also affect the assessment of UEH. In this study, the matter element analysis is superior to some available models (the fuzzy synthetic assessment model and the comprehensive index method) due to the fact that it did not divided the ecosystem health artificially into several grades and resulted in more objectivity and accuracy [22]. In addition, the complicated computation processes limited the application of some mathematical models such as maximum information entropy method [23]. The theory of matter element analysis is easy to understand and can be calculated in basic software such as Matlab or Excel, which is much simpler than other methods.

## 5. Conclusions

Assessment of the urban ecosystem health can help to identify the conditions and limiting factors in an urban ecosystem, which can further help the government and residents to propose reasonable management strategies. In this research, the Vigor-Organization-Resilience-Service-Function-Population health framework and matter element analysis were applied to assess the UEH status of each city in Jiangsu province during 2000–2014. The results revealed that the strengths and weaknesses of all the cities show regional characteristics, and the UEH status of SJ and CJ was better than that of NJ. Meanwhile, the result indicated the obstacle degree to urban ecosystem health was successively organization > vigor > population health > service function > resilience during 2000–2014, which demonstrated that it is worth to pay attention to the decline of the ecosystem organization in some cities. The results also showed that the matter element analysis was more objective and accurate than some traditional methods (fuzzy synthetic assessment model) and more simple than some mathematic methods (maximum information entropy method).

## Figures and Tables

**Table 1:** Characteristics of each city in Jiangsu Province in 2014.

Characteristic	Region												
	NJ	WX	XZ	CZ	SZ	NT	LYG	HA	YC	YZ	ZJ	TZ	SQ
X1	10.77	12.65	5.78	10.45	13.01	7.75	4.44	5.09	5.31	8.27	10.28	7.27	4.01
X2	10.1	8.2	10.6	10.1	8.4	10.6	10.2	11	10.9	11	10.9	10.8	10.8
X3	0.55	0.46	0.46	0.46	0.46	0.505	0.46	0.46	0.46	0.505	0.55	0.46	0.46
X4	4.26	4.17	2.41	3.95	4.67	3.34	2.36	2.58	2.59	3.03	3.58	3.13	2.04
X5	213.9	188.8	107.8	156.6	527.8	149.8	62	77.9	68.1	90.2	84.2	61	43.2
X6	44.1	42.9	43.3	43	42.2	42.6	40	40.9	40.5	43.6	42.5	40.7	42.3
X7	1247	1405	733	1074	1225	692	585	484	427	679	826	802	568
X8	4.09	2.54	20.32	2.7	3.08	−0.59	15.51	12.48	4.5	2.33	1.53	1.21	18.93
X9	56.5	48.4	45.2	48.0	48.4	44.2	41.4	44.1	40.8	42.9	46.1	43.4	38.9
X10	56.4	38.8	34.8	37.2	35.9	31.2	36.2	40.5	37.7	36.4	41.0	33.9	27.7
X11	10.2	9.4	9.5	8.9	10.5	9.7	13.3	12.6	10.9	8.0	8.5	8.2	10.9
X12	6.6	16	18.8	16.1	4	18.1	27.1	23.6	24.1	19.3	22.2	24.6	20.8
X13	95.2	99.3	99	100	99.5	99.4	98.1	100	92.6	98.3	98.5	97.4	94.7
X14	95.3	96.8	92.7	95	95.7	92.8	84.1	91	89.2	93.7	92.8	89.5	93.5
X15	91.9	91.1	99	98.2	96.7	98.3	93.7	99.5	93.9	92.3	98.6	98.3	94
X16	3.5	3.4	3.7	3.2	3.9	3.7	2.9	3.1	2.8	2.9	3.1	3.4	2.9
X17	52.1	57.7	65.6	63.8	72.6	70.8	69.4	60.8	74	65.5	65.9	65.8	63.8
X18	53.8	56.2	56.7	52.6	54	58	53.3	53.9	51.3	54.6	54.8	53.3	53
X19	15	14.8	16.2	13.2	15.2	16.8	14.2	13.8	12	18	18.7	9.5	13.8
X20	36.3	44.8	40.5	43.7	44	46.3	45.3	43.6	42.7	42.1	44.2	48	46.5
X21	22.2	25.2	25.3	25.5	28.1	29.2	21.4	20.9	20.1	21.7	24.2	24.3	27.7
X22	11.5	13.3	9.8	9	9.2	10.2	2.9	11.2	4.2	9.5	6.8	4.2	6.4
X23	2.5	2.12	2.4	2.14	2.4	2.3	2.6	2.6	2.4	2.3	2.12	2.2	2.5
X24	26	28.7	30.8	28.3	26.9	29	32.3	31.3	31.9	30.9	28.5	29.1	36.1
X25	3	4.4	4.4	3.5	4.4	4.4	4.4	2.9	4.4	4.4	2.8	3.4	4.4
X26	47.6	47.7	50	45.8	49.9	46.8	37.1	47.1	46.6	39	40.2	42.1	41.6
X27	980	176	159	231	198	111	86	139	78	181	266	106	36

Note: X1–X27 was the initial value of indicators in each city in 2014. The details of X1–X27 were shown in Table 2. NJ: Nanjing; WX: Wuxi; XZ: Xuzhou; CZ: Changzhou; SZ: Suzhou; NT: Nantong; LYG: Lianyungang; HA: Huai’an; YC: Yancheng; YZ: Yangzhou; ZJ: Zhenjiang; TZ: Taizhou; SQ: Suqian.

**Table 2:** Weight and assessment indicator system of UEH.

Component	Indicator	Weight	Reference
Vigor	X1 Per capita GDP (10,000 CNY)	0.0221	[12,14,15]
	X2 GDP growth (%)	0.0253	[3,15]
	X3 Energy consumption per 10,000 CNY of GDP(ton of SCE/10,000 CNY)	0.0474	[14,15,32]
	X4 Per capita annual disposable income of urban households (10,000 CNY)	0.0331	[14,15,32]
	X5 Actual use of foreign capital (100 Million CNY)	0.0179	[25]
	X6 Green covered area as of completed area (%)	0.0303	[12,14]
Organization	X7 Population density of urban area (persons/km^2^)	0.0467	[12,14,15]
	X8 Natural growth rate of population (‰)	0.0472	[3,12,23]
	X9 Tertiary industry accounted for the proportion of GDP (%)	0.0590	[14,15,32]
	X10 Proportion of tertiary industry employment (%)	0.0392	[25]
	X11 Fiscal revenue accounted for the proportion of GDP (%)	0.0352	[3,25]
	X12 Growth rate of total investment in fixed assets (%)	0.0378	[23,25]
Resilience	X13 Attainment rate of the industrial wastewater discharged (%)	0.0602	[12,15]
	X14 Urban wastewater treatment rate (%)	0.0438	[12,15]
	X15 Common industrial solid wastes comprehensively utilized (%)	0.0348	[12,15]
	X16 Total investment in the treatment of environmental pollution as percent GDP (%)	0.0660	[14,15,32]
Service function	X17 Proportion of days of air quality equal to or above grade II in the whole year (%)	0.0396	[14,15]
	X18 Urban environmental noise (dB)	0.0215	[25]
	X19 Per capita area of parks and green land (m^2^)	0.0332	[12,14,15]
	X20 Per capita urban residential area (m^2^)	0.0296	[12,15,23]
	X21 Per capita area of paved roads in city (m^2^)	0.0298	[12,14,15]
	X22 Number of public transportation vehicles per 10,000 population in city (unit)	0.0212	[14,15]
	X23 Registered urban unemployment rate (%)	0.0419	[12,14,15]
Population health	X24 Engel’s coefficient of urban households (%)	0.0524	[14,15,23]
	X25 Under 5 mortality rate (‰)	0.0360	[25]
	X26 Number of beds of hospitals and health centers per 10,000 population (bed)	0.0282	[14,15,23]
	X27 Number of students’ enrollment of regular institutions of higher education per 10,000 population (10,000 persons)	0.0208	[3,14,15]

**Table 3:** The classical domain and joint domain of UEH.

Indicators	Grade					Reference
	Poor	General	Medium	Good	Excellent	
X1	0–2	2–4	4–8	8–12	12–16	[12,14]
X2	0–4	4–8	8–12	12–16	16–30	[32,34]
X3	1.5–2	1.2–1.5	0.9–1.2	0.6–0.9	0–0.6	[14,32,34]
X4	0–0.8	0.8–1.5	1.5–2.5	2.5–3.5	3.5–5	[14,32,34]
X5	0–100	100–200	200–300	300–400	400–600	[25]
X6	0–20	20–30	30–40	40–50	50–80	[14,32]
X7	1300–1500	1100–1300	900–1100	700–900	400–700	[12,32]
X8	15–25	12–15	9–12	6–9	2–6	Experts’ opinion
X9	0–20	20–30	30–40	40–50	50–80	[14,32]
X10	0–20	20–30	30–40	40–50	50–80	[25]
X11	0–5	5–10	10–15	15–20	20–25	Experts’ opinion
X12	0–10	10–20	20–30	30–40	40–50	Experts’ opinion
X13	50–75	75–80	80–85	85–95	95–100	[12,14,32]
X14	40–50	50–70	70–80	80–90	90–100	[14,32,34]
X15	40–50	50–70	70–80	80–90	90–100	[12,32,34]
X16	0–1.5	1.5–2.5	2.5–3.5	3.5–4.5	4.5–6	[14,25,34]
X17	0–20	20–40	40–60	60–80	80–100	[14,32]
X18	85–100	70–85	55–70	45–55	30–45	[25]
X19	0–4	4–7	7–10	10–16	16–20	[12,14]
X20	0–15	15–25	25–35	35–45	45–55	[12,25]
X21	0–5	5–10	10–20	20–25	25–30	[12,14]
X22	0–5	5–10	10–15	15–20	20–25	[14,25]
X23	20–25	15–20	10–15	5–10	0–5	[12,14]
X24	40–60	35–40	30–35	25–30	0–25	[14,32,34]
X25	16–20	12–16	8–12	4–8	0–4	Experts’ opinion
X26	0–100	100–300	300–500	500–800	800–1200	[14,25]
X27	0–50	50–150	150–300	300–600	600–1000	[25]

**Table 4:** UEH grade of each indicator of 13 cities in Jiangsu Province.

Region	Urban Ecosystem Health Grade of Each Index (2014)																										
	X1	X2	X3	X4	X5	X6	X7	X8	X9	X10	X11	X12	X13	X14	X15	X16	X17	X18	X19	X20	X21	X22	X23	X24	X25	X26	X27
Nanjing	IV	III	V	V	III	IV	II	V	V	V	III	I	V	V	V	III	III	IV	IV	IV	IV	III	V	IV	V	I	V
Wuxi	V	III	V	V	II	IV	I	V	IV	III	II	II	V	V	V	III	III	III	IV	IV	V	III	V	IV	IV	I	III
Xuzhou	III	III	V	III	II	IV	IV	I	IV	III	II	II	V	V	V	IV	IV	III	V	IV	V	II	V	III	IV	I	III
Changzhou	IV	III	V	V	II	IV	III	V	IV	III	II	II	V	V	V	III	IV	IV	IV	IV	V	II	V	IV	V	I	III
Suzhou	V	III	V	V	V	IV	II	V	IV	III	III	I	V	V	V	IV	IV	IV	IV	IV	V	II	V	IV	II	I	III
Natong	III	III	V	IV	II	IV	V	I	IV	III	II	II	V	V	V	IV	IV	III	V	V	V	III	V	IV	II	I	II
Lianyungang	III	III	V	III	I	IV	V	II	IV	III	III	III	V	IV	V	III	IV	IV	IV	V	IV	I	V	III	II	I	II
Huai’an	III	III	V	IV	I	IV	V	V	IV	IV	III	III	V	V	V	III	IV	IV	IV	IV	IV	III	V	III	V	I	II
Yancheng	III	III	V	IV	I	IV	V	V	IV	III	III	III	V	IV	V	III	IV	IV	IV	IV	IV	I	V	III	II	I	II
Yangzhou	IV	III	V	IV	I	IV	V	V	IV	III	II	II	V	V	V	III	IV	IV	V	IV	IV	II	V	III	II	I	III
Zhenjiang	IV	III	V	V	I	IV	IV	V	IV	IV	II	III	V	V	V	III	IV	IV	V	IV	IV	II	V	IV	V	I	III
Taizhou	III	III	V	IV	I	IV	IV	V	IV	III	II	III	V	IV	V	III	IV	IV	III	V	IV	I	V	IV	V	I	II
Suqian	III	III	V	III	I	IV	V	I	III	II	III	III	V	V	V	III	IV	IV	IV	V	V	II	V	II	II	I	I

Note: I, refers to poor grade; II, general grade; III, medium grade; IV, good grade; and V, excellent grade.

**Table 5:** Synthetically correlation degree and matter grade of UEH in 2000, 2005, 2010, and 2014.

Region	Synthetically Correlation Degree (2014)					2014	2010	2005	2000
	Poor	General	Medium	Good	Excellent	Grade			
Nanjing	–0.4549	–0.3786	–0.2670	–0.1723	–0.1439	Excellent	Excellent	Good	Good
Wuxi	–0.5458	–0.4722	–0.3789	–0.2918	–0.2063	Excellent	Excellent	Medium	General
Xuzhou	–0.5132	–0.4357	–0.3253	–0.2486	–0.2369	Excellent	Excellent	Medium	General
Changzhou	–0.5625	–0.4509	–0.3529	–0.2815	–0.1946	Excellent	Excellent	Medium	General
Suzhou	–0.5809	–0.5168	–0.4400	–0.3267	–0.1835	Excellent	Excellent	Medium	Medium
Nantong	–0.5925	–0.4873	–0.3962	–0.3001	–0.1790	Excellent	Excellent	Medium	General
Lianyungang	–0.4896	–0.4139	–0.2896	–0.2320	–0.2507	Good	Excellent	Medium	General
Huaian	–0.5291	–0.4309	–0.2955	–0.2621	–0.2163	Excellent	Excellent	Medium	General
Yancheng	–0.5228	–0.4296	–0.3044	–0.2214	–0.2252	Good	Good	Excellent	General
Yangzhou	–0.5498	–0.4461	–0.3294	–0.2428	–0.2020	Excellent	Excellent	Good	General
Zhenjiang	–0.5705	–0.4832	–0.3701	–0.2653	–0.1798	Excellent	Excellent	Medium	General
Taizhou	–0.5405	–0.4552	–0.3386	–0.2522	–0.2184	Excellent	Excellent	Medium	General
Suqian	–0.4783	–0.4113	–0.3375	–0.2975	–0.2581	Excellent	Excellent	Excellent	Poor

**Table 6:** The health level and area statistics of UEH in Jiangsu Province.

UEH Level	2000		2005		2010		2014	
	Area (km^2^)	Proportion (%)	Area (km^2^)	Proportion (%)	Area (km^2^)	Proportion (%)	Area (km^2^)	Proportion (%)
Excellent	0	0.0	25,444	24.8	85,711	83.5	78,265	76.3
Good	6597	6.4	13,231	12.9	16,889	16.5	24,335	23.7
Medium	8488	8.3	63,814	62.3	0	0.00	0	0.00
General	78,960	77.0	0	0.00	0	0.00	0	0.00
Poor	8555	8.3	0	0.00	0	0.00	0	0.00
